# Proceedings of Veterinary Medical Societies

**Published:** 1888-01

**Authors:** 


### PROCEEDINGS VETERINARY SOCIETIES.

I. UNITED STATES VETERINARY MEDICAL ASSOCIATION.*—This Association held its twenty-fourth annual meeting at the American Veterinary College in New York on Tuesday, September 19th.

The Comitia Minora was called to order at 10:30 A. M. by the President, Dr. Liautard—present Drs. Liautard, Huidekoper, Zuill Dixon, L. McLean and Michener. Ten graduates and one non-graduate, candidates for admission to membership were reported favorably. The regular meeting was called at half past eleven, President Dr. Liautard in the chair; over forty members were present.

The minutes of the last meeting were read and approved, with the correction of Dr. Salmon's election as an active member—the Secretary having recorded him as an honorary member. The minutes of the Comitia Minora were read and approved.

The committee on Intelligence and Education made a short re

*This report was mislaid by printer at last issue of the JOURNAL.

port through the Chairman, Dr. Huidekoper, including a clear statement from Dr. Paquin of Missouri on the demands of the West for a more complete education in anatomy and in the diseases of animals other than the horse.

Dr. Hoskins, Chairman of the Committee for Securing a Uniform Education, reported progress.

The Committee on Diseases, Dr. Zuill, Chairman, reported at length on the prevalence of Hog Cholera, Anthrax, Glanders, Tuberculous, Contagious Pleuro-Pneumonia, Chicken Cholera and Cerebro-Spinal Meningitis, which latter, while not contagious, had existed to some extent in epizootic form.

The Prize Committee was unable to decide the merits of the two papers represented. Dr. L. H. Howard was added to the committee to fill the place of Dr. Meyer, absent, and the papers referred back for a report at this meeting.

The Committee on Army Legislation presented a printed bill and progress. The Committee was continued. By vote of the Association the Secretary cast an affirmative vote for the election of the following gentlemen for admission to membership.

Drs. T. W. Mayer, Werner, Turner, Lamberton, Barrow, Strange, C. C. McLean, Blank, Connell, Sellers and Farnsworth.

The Treasurer's accounts were audited and his report received.

A large number of new candidates were presented for membership.

The Prize Committee reported that they did not consider either essay as deserving of the prize. On motion of Dr. McLean, seconded by Dr. Clement, the report was received.

The election of officers for the ensuing year resulted as follows: *President*, Dr. R. S. Huidekoper; *Vice-President*, Dr. J. C. Meyer, Jr.; *Secretary*, Dr. C. B. Michener; *Treasurer*, Dr. Jas. L. Robertson; *Board of Censors*, Drs. Dixon, Lyman, Hoskins, Zuill, L. McLean, Ross and Bryden.

Dr. Liautard presented the new president, Dr. Huidekoper, who took the chair.

Dr. Liautard presented several forms of operating tables for inspection and criticism of the members.

Dr. Michener offered the alteration of Section I., Art. IV. of the By-Laws, which was adopted. *None but Graduates* are eligible for membership of this Association. By vote the papers offered for prizes were considered in general meeting. A motion was carried that the papers be balloted for and the prize awarded to the one receiving the largest number of votes. The President notified the meeting that this action could not influence the granting of the additional prize offered by Dr. Liautard. The ballot resulted in the largest number of votes being cast for "Trianon"—Dr. John Wende of Buffalo, N. Y.

Dr. McLean gave notice that at the next meeting he would offer an alteration to Article X. Chapter VII. of the Constitution, making it read as follows: "Hereafter, prizes will only be given to the best original paper, read and defended by the author at the annual meeting."

Bills presented by the Treasurer were ordered paid.

On motion of Dr. Martinet, it was decided to hold the semi-annual meeting next March in Baltimore, Maryland.

A desultory discussion on Cerebro-spinal meningitis occupied some time. Owing to the time having been entirely occupied by discussions of the above business the papers prepared for the meeting were not read.

In the evening a handsome banquet was held at Clark's for which the thanks of the Association is due to the local Committee of Arrangements.

The following toasts were offered and responded to: "The President of the United States," Dr. Bryden; "Our Profession," Dr. Liautard; "Our Sister Profession," Dr. Raymond; "The Bureau of Animal Industry," Dr. Michener; "Our Legislature," Hon. J. A. Cantor; "The Press," Dr. Hummel; "The Ladies," Dr. Pendry.

II. NATIONAL VETERINARY SANITARY ASSOCIATION.—The third annual meeting of this society was held at Kansas City, Mo., Oct. 31 and Nov. 1 and 2.

President J. L. Brush, of Colorado, was absent, and First Vice-President Dr. James Hopkins, of Wyoming, occupied the Chair. Dr. Paquin, of Missouri, Secretary.

The following members were present: Hon. Norman J. Colman, Commissioner of Agriculture; Dr. D. E. Salmon, Chief of the Bureau of Animal Industry; Dr. J. D. Hopkins, Territorial Veterinarian of Wyoming; Dr. Paul Paquin, State Veterinarian of Missouri; Dr. A. A. Holcomb, State Veterinarian of Kansas; Dr. Julius Gerth, Jr., State Veterinarian of Nebraska; Dr. A. J. Chandler, State Veterinarian of Arizona; Dr. Herbert Holloway, Territorial Veterinarian of Montana; Dr. M. Stalker, State Veterinarian of Iowa; Dr. W. Williams, Assistant State Veterinarian of Illinois; Prof. Morrow, of the University of Illinois; Dr. T. E. White, Sedalia, Mo.; Mr. S. S. Hinds, Presid enLive Stock Com., of Michigan; Mr. Woodburn, member of Michigan Live Stock Com.; Dr. H. B. Adair, Society Against Cruelty to Animals, Kansas City; Dr. Gadsden, Philadelphia, Pa.; Dr. F. Allen, Emporia, Kansas; Dr. M. D. Lewis, Rocheport, Mo.; A. S. Mercer, editor *Northwestern Live Stock Journal*, Wyoming; Mr. Pearson, Chairman Illinois Live Stock Commission; Messrs. McChesney and Wilson, members of the same board; F. W. Smith, State Board of Agriculture, of Missouri. Letters expressing regret for non-attendance, were received from the following gentlemen and ordered on file: Dr. Alex. Liautard, American Veterinary College; Dr. A. H. Baker, Chicago Veterinary College; Dr. A. Smith, Ontario Veterinary College; Dr. Osler, Pennsylvania University; Dr. W. L. Zuill, Veterinary Department Pennsylvania University; Dr. T. Wesley Mills, McGill College, Canada; Dr. McIntosh, Illinois Industrial University; Dr. Comstock College of Veterinary Surgeons New York City, N. Y.; Dr. V. T. Atkinson, State Veterinarian of Wisconsin; Dr. Miller, of Camden, New Jersey; Dr. Butler, of Ohio; Dr. Robinson, of Virginia and others.

The Association held four sessions, three of which were consumed almost entirely in the discussion of contagious Pleuro-Pneumonia and the sanitary regulations which the United States and various States and Territories should adopt to suppress it. A very lively discussion arose after the announcement by the Commissioner of Agriculture that the quarantines of the Bureau of Animal Industry against Cook Co., Illinois, would be raised about the 1st of December, and that the State of Illinois also intended to give freedom to the cattle of that part of her land. The Territorial Veterinary Sanitarians were unanimous in opposing this intention, and made their reasons known freely before the Society. The animated debate increased when the following resolution was read by the Secretary and seconded by Dr. C. J. Alloway, of Dakota:

*Resolved*, That while we are well pleased with the report of the Commissioner of Agriculture for the good work done against contagious Pleuro-Pneumonia at Chicago, we think it unwise to remove quarantine against Cook County before next Spring, and respectfully recommend that it be continued till then.

This resolution, which was meant to bring out the sentiment of the State and Territorial officials on the subject, had the desired effect. While the committee on resolutions, Drs. Holcomb Gerth and Alloway, made two reports, a majority report against it and a minority report in favor of it, it became apparent that the majority desired a continuance of the restriction against Cook Co., Ill., for at least some time after the 1st of December, because if the reverse occurred the Territories and States would respectively feel forced in self-defense, to keep up cumbersome restrictions against Illinois. The Chief of the Bureau of Animal Industry, was desirous of guarding the interest of all the States and Territories alike, and spoke in a manner to give assurance to all the representatives that they would keep close guard on Cook County that none need fear it, as it was free from contagious Pleuro-Pneumonia. The short time spent by the Bureau and the Illinois Commission in stamping out Pleuro-Pneumonia in Chicago, makes other States fear that it is not yet truly exterminated. But we must admit that the officers did wonderfully thorough and rapid work of disinfection and slaughtering—better probably, than was ever done in this country, if not in any country, if we may judge by history and official reports.

After a long and interesting discussion the resolution favored especially by Drs. Hopkins, Alloway and Chandler, was finally tabled, some members in its favor not voting.

Texas fever was then discussed. It appeared that this year the States of Missouri, Illinois, Nebraska and Kansas had suffered the most, with Missouri in the lead. This State has unfenced railways, and many serious outbreaks occurred on those lines among cattle grazing about them and over them. The East St. Louis stock yards had also distributed the germs of the disease to a considerable extent, whilst Kansas City stock yards, with its efficient sanitary system, under the direction of Dr. A. A. Holcomb, had caused but very little trouble—a great improvement on the preceding years. Kansas and Nebraska, however, had apparently received the malady from these yards a few times.

*Maladie du Coit*, as it appeared in Illinois, was also slightly discussed. A few of the members had been on the ground to study the disease and gave interesting accounts of what they had seen. A very valuable article had been prepared on this subject by Dr. W. W. Williams, of Bloomington, Ill., who had charge of the diseased stock, but unfortunately it was crowded out by pressure of business. We trust that in the future more time will be given to papers of this nature, and that more papers will be prepared for the Association, which is a body destined to be of more and more utility and power in dealing with contagious diseases of live stock and influencing wiser legislation for protection against them.

All the members were delegates also to the Cattlemen's Convention, and had a very pleasant time during their stay in Kansas City, which with its cable cars, elevated railways, electric street trains, is probably the busiest city of its size anywhere in the United States.

The officers for the ensuing year are as follows:

*President*, Dr. A. A. Holcombe, of Kansas City.

*First Vice-President*, (re-elected), Dr. James Hopkins, of Wyoming.

*Second Vice-President*, Dr. C. J. Alloway, of Dakota.

*Secretary*, (re-elected), Dr. Paul Paquin, of Missouri.

*Assistant Secretary*, Dr. Herbert Holloway, of Montana.

III. ONTARIO VETERINARY COLLEGE.—The matriculation examinations for the Ontario Veterinary College were held on October 24th and following days. A great many candidates presented themselves, the great majority being from the United States and Ontario, some coming from the Province of Quebec, the Maritime Provinces, and different parts of the Old World.

The formal opening of the session of 1887-88 took place on October 26th, in Richmond Hall, when Professor Smith, the Principal of the College, delivered the opening lecture. Richmond Hall was well filled by students to listen to this lecture, which was full of words of welcome, encouragement and good cheer.

In many ways does the College show signs of vigorous growth; specially in the matters of accommodation, attendance and teaching arrangements. As to accommodations, the hall which was used last winter was then so crowded as to be uncomfortable. This being the case the determination had been arrived at to erect new college buildings, containing the amplest halls and class-rooms necessary. The strikes in the building trades during the past summer unfortunately rendered this impossible. Fortunately Richmond Hall, conveniently situated in regard to the other college buildings, was secured. This hall will seat four hundred people, and it makes a very pleasant auditorium.

The vacancy in the staff caused by the lamentable death of Dr. Barrett has been filled by the appointment of Dr. Peters to the chair of Physiology. Dr. Peters is a gold medallist of Toronto University, a zealous and experienced teacher, and a popular lecturer. He holds a position in the University Medical School. Dr. John Cavan has been given the chair of Pathology, and is expected to do much for the advancement of this branch of study. He has paid a great amount of attention to the subject, both in this country and also in Germany, where he studied under the best teachers. He will devote a considerable portion of his time to this department, not only giving the results of the labors of the best pathologists, but taking up, and endeavoring to work for the College and the students, some of the unsolved problems on the works of pathology.

Professor Ramsay Wright, whose lectures on Bacteriology created so much interest last spring, will give a similar course this session.

